# Emergency department utilisation and treatment for trauma-related presentations of adolescents aged 16–18: a retrospective cross-sectional study

**DOI:** 10.1186/s12873-024-00945-8

**Published:** 2024-02-27

**Authors:** Lara Brockhus, Elias Hofmann, Kristina Keitel, Martina Bärtsch, Martin Müller, Jolanta Klukowska-Rötzler

**Affiliations:** 1 Department of Emergency Medicine, Inselspital, Bern University Hospital, Bern University, Bern, Switzerland; 2 Department of Paediatric Emergency Medicine, Inselspital, University Children’s Hospital, Bern University, 3010 Bern, Switzerland; 3 Department of Paediatrics, Inselspital, University Children’s Hospital, Bern University, 3010 Bern, Switzerland

**Keywords:** Adolescents, Trauma, Sex comparison

## Abstract

**Background:**

A recent study conducted at our tertiary hospital emergency department (ED) reviewed ED consultations and found that adolescents aged 16–18 years present significantly more often for trauma and psychiatric problems than adults over 18 years. Accidental injuries are one of the greatest health risks for children and adolescents. In view of the increased vulnerability of the adolescent population, this study aimed to further analyse trauma-related presentations in adolescents.

**Methods:**

We conducted a single-centre, retrospective, cross-sectional study of all adolescent trauma patients aged 16 to 18 years presenting to the adult ED at the University Hospital (Inselspital) in Bern, Switzerland, from January 2013 to July 2017. We analysed presentation data as well as inpatient treatment and cost-related data. Data of female and male patients were compared by univariable analysis. A comparison group was formed consisting of 200 randomly chosen patients aged 19–25 years old with the same presentation characteristics. Predictive factors for surgical treatment were obtained by multivariable analysis.

**Results:**

The study population included a total of 1,626 adolescent patients aged 16–18 years. The predominant causes for ED presentation were consistent within case and comparison groups for sex and age and were sports accidents, falls and violence. Male patients were more likely to need surgical treatment (OR 1.8 [95% CI: 1.2–2.5], *p* = 0.001) and consequently inpatient treatment (OR 1.5 [95% CI: 1.1–2.1], *p* = 0.01), associated with higher costs (median 792 Swiss francs [IQR: 491-1,598]). Other independent risk factors for surgical treatment were violence-related visits (OR 2.1 [95% CI: 1.3–3.5, *p* = 0.004]) and trauma to the upper extremities (OR 2.02 [95% CI: 1.5–2.8], *p* < 0.001). Night shift (OR 0.56 [95% CI: 0.37–0.86], 0.008) and walk-in consultations (OR 0.3 [95% CI: 0.2; 0.4, < 0.001] were preventive factors for surgical treatment.

**Conclusions:**

Male adolescents account for the majority of emergency visits and appear to be at higher risk for accidents as well as for surgical treatment and/or inpatient admission due to sports accidents or injuries from violence. We suggest that further preventive measures and recommendations should be implemented and that these should focus on sport activities and injuries from violence.

**Supplementary Information:**

The online version contains supplementary material available at 10.1186/s12873-024-00945-8.

## Introduction

Accidental injuries are one of the greatest health risks for children and adolescents. It is estimated internationally that nearly one million children and adolescents die each year due to injuries caused by accidents [1]. This is almost a third of the number of children who die due to poor nutrition and hunger—one of the top ten problems in global health and which, according to the estimate of the UNICEF (United Nations Children’s Fund), affects around 3 million children per year [2]. According to the WHO (World Health Organisation), accidents are by far the largest cause of DALYs (Disability Adjusted Life Years) among 15–19 year olds in Europe and worldwide [3]. In Switzerland, about 100 children and adolescents per million inhabitants die in accidents every year [4].

A much larger number of minors are injured in non-fatal accidents—mainly in sports, at home, and during leisure activities [4]. In Europe alone, accidental injuries to children and adolescents cause about 5.4 million inpatient hospital admissions and 68.7 million emergency room treatments every year [5]. Age and sex differences are evident in many data sources [6].

Hospitals are regularly confronted with vulnerable patient groups, such as children and adolescents—especially in the ED (Emergency Department). The adult ED at the University Hospital of Bern (Inselspital) in Switzerland treats patients aged 16 years and over. Consequently, adolescents as young as 16 may be treated in the adult ED, while younger patients are referred to the paediatric ED nearby.

The age limit of 16 years was set historically, and there is no scientific explanation for this age cut off. 16–18 year olds are an age group in transition from paediatrics to adult medicine and are often treated as “small adults”, even though they differ drastically in physiology and psychology [7, 8]. They are at a crucial point of development, which leads to unique challenges for ED care. The legal and ethical circumstances for consent and confidentiality are changing. These complex issues need to be handled sensitively and appropriately, and must both involve the family and respect the growing autonomy of the young adult [9]. The transition from paediatric to adult care is a critical step for people with chronic conditions and is becoming increasingly significant as the mortality in this group has decreased in recent decades [10]. It should therefore be planned early and based more on biopsychosocial development than on chronological age [10]. Continuity of care and adherence to treatment are of the utmost importance, and this can be particularly challenging in emergencies.

There is little information on ED utilisation by adolescents in Switzerland. Studying this population can therefore provide valuable data for health policy, practice, and improvements in emergency care for adolescents. A review study conducted by our research department [11] examined this specific patient group and analysed the demographic data. For the period from 2013 to 2017, we analysed ED presentations of adolescents between 16 and 18 years of age and their main reasons for presenting. Furthermore, we compared the findings with results from adult patients older than 18 years. We found that 16–18 year olds presented significantly more often for trauma and psychiatric problems than persons over 18 years [9]. On the basis of this review paper, we decided to further investigate the subgroups of psychiatric and traumatic ED presentations of adolescents for the same time period (2013–2017), as it was important to achieve more detailed understanding of this vulnerable patient population [12].

This study aims to provide an extended overview of demographic data in trauma-related ED presentations of adolescent patients and includes a comparison to adult patients aged 19–25. Primary endpoints are assessed—including sex, age, injury mechanisms, injured body parts, need for surgical intervention, inpatient treatment, and ICU-stays. The secondary endpoint was to determine independent risk factors for increases in the rate of surgical treatment of the sustained injuries. Our broader objective was to derive measures for treatment and prevention from the results obtained.

## Methods

### Study design and setting

We conducted a retrospective study of anonymised data. We analysed trauma-related presentations by adolescent patients aged 16 to 18 years at our tertiary adult ED at the University Hospital in Bern (Inselspital). Our analysis covered the patients’ medical history and clinical data for the period from January 2013 to July 2017. The results obtained were compared with those from a group of 200 randomly selected trauma cases aged between 19 and 25 years who presented to our ED in the same time period. This patient group had been selected as a comparison group in a prior study conducted at our ED describing presentations of adolescent patients and corresponded to approximately 10% of the total population in this study of 1,626 [11]. The present study was in compliance with the reporting guideline for Strengthening the Reporting of Observational Studies in Epidemiology (STROBE) [13] and was conducted without an official study protocol.

### Ethical considerations

For this descriptive study, we obtained permission from the Bern Cantonal Ethics Committee (No 2022 − 00454).

### Participants and data sources

**Fig. 1:**
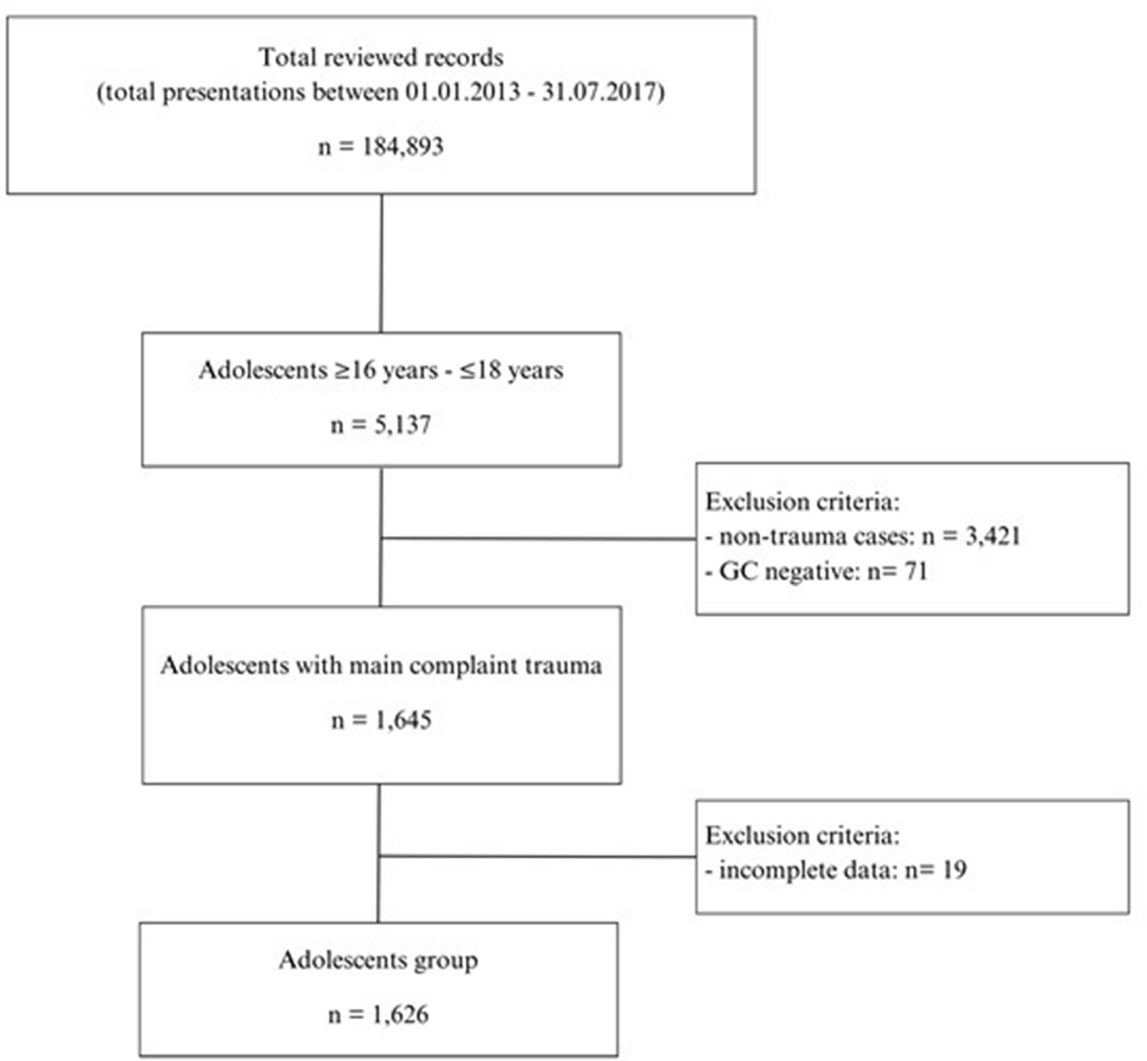
Flow chart of selection of medical records

In order to form the main study group (adolescents), we searched the ED electronic medical record system (E.care BVBA, ED 2.1.3.0, Turnhout, Belgium) and identified all trauma-related presentations to the ED between January 2013 and July 2017. The data base was searched for all presentations that were managed as trauma cases. Adults over 18 years old were excluded by the definition of the study population (adolescents ≤ 18 years old) and children under 16 years (< 16 years) were excluded as they are admitted to our paediatric ED nearby (Fig. 1). All non-trauma related presentations were excluded as well. The comparison group included 200 randomly chosen cases from trauma patients aged 19–25 years presenting to our ED (comparison group). These 200 cases represented more than 10% of all adolescents’ cases (*n* = 1,626), which was a sufficient size for statistical comparison. Cases with missing clinical or administrative data were excluded. No individual informed consent was obtained, yet patients who explicitly refused general consent (consent to contribute patient data for current or future research purposes [14]) were excluded from the study.

### Study size

The study size was not actively defined but depended on the given data in accordance with our search terms and included all cases of trauma related ED-presentations in adolescents presenting to our tertiary ED.

### Quantitative variables

The data collection resulted in an extensive data pool. We conducted a descriptive statistical analysis of the data to highlight demographic aspects of every patient in our study population. The Inselspital adult ED performs triaging for every patient, using the mandatory Swiss Emergency Triage Scale, even after the treatment has started (for example in polytrauma patients). On arrival, patients are questioned (short medical history) and examined (vital parameters) by specialised nurses, in order to define the patient’s main complaint and the urgency for treatment ((1) life-threatening situations (life/limb-threatening situations—immediate treatment); (2) highly urgent situations (potentially life-threatening situations—assessment and treatment within 20 min); (3) urgent situations (assessment and treatment within 120 min); (4) semi-urgent and (5) non-urgent situations. The assessment includes coding of the main complaint. For our study, we included patients who were triaged with “trauma” as the main reason for presentation. We exclusively sampled anonymised ED presentations, so that multiple visits of one patient might have been included separately.

### Studied characteristics

We analysed the following ED presentation-related variables (primary endpoints): sex, age, triage category, type/time of ED admission and discharge, mechanism of injury, injured body region. Furthermore, we wanted to address extended data points of this population such as inpatient treatment, ICU stays, in-hospital mortality, and costs (total costs of ED presentation, in case of inpatient treatment costs of hospitalisation).

#### Risk factor analysis

For quantifying injury severity, the following variables were collected: (i) treatment in the care room for emergency trauma, (ii) diagnostic work-up (i.e. need for CT scans) (iii) need for inpatient treatment, (iv) type of treatment, in particular surgical treatment, (v) ICU stay, (vi) in-hospital mortality, and (vii) ED as well as hospital costs. It is emphasised that there was no standardised questionnaire for the patient history.

The secondary endpoint was to identify independent risk factors that led to an increased risk of surgery in patients aged 16–18 years (see 2.7. Statistical analysis).

### Statistical analysis

For statistical analysis, we employed STATA® 16.1 (StataCorp, The College Station, Texas, USA). Categorical variables were presented as frequencies (proportions). Categorical variables were compared using chi square tests and continuous variables using the Wilcoxon rank sum test. Univariable logistic regression analyses were used to obtain the effect sizes, i.e. odds ratio (OR) with 95% confidence interval (CI), in order to compare males with females, adolescents with young adults and surgical vs. conservative treatment.

A multivariable analysis was conducted to determine factors that favour surgical treatment. Variables that showed at least a weak association (*p* < 0.20) for an association with surgical treatment were considered as potential confounders [15, 16]. These included sex, presentation during night shift (10 pm − 6 am) (protective), injuries to the upper extremities, high urgency triaging, walk-in patients (protective) and violence. The final model was determined by performing stepwise backward logistic regression (*p* < 0.05) [17, 18]. Categorical variables were binarised to determine individual effects (shift of presentation, injured body part, injury mechanics) and in order to achieve a parsimonious model. Triage and type of admission were grouped for regression analysis to reduce the number of covariates (i.e. “documented high-urgent” includes triage groups 1 and 2, “documented walk-in” includes all walk-in patients compared to all other means of presentation combined).

Lastly, variables identified through multivariable analysis within the case group were incorporated in a multivariable logistic regression model including the control group (aged 19–25 years), in order to predict surgical treatment and to analyse whether the predictors were similar in the two study groups.

We sought to find largely independent variables for the increase in the risk of surgery.

## Results

### Participants

We reviewed a total of 184,893 presentations to the adult ED at the University Hospital of Bern from January 2013 to July 2017. 177,732 adults > 18 years old and 2,024 children < 16 years old were excluded, thus resulting in 5,137 cases involving adolescents aged ≥ 16 years - ≤18 years. After excluding non-traumatic presentations (*n* = 3,421) and cases with negative general consent (*n* = 71) for resource analysis, another 19 patients were excluded due to missing administrative data. We finally included 1,626 cases of adolescents presenting with trauma (Fig. 1).

To form the control group, a total of 25,063 cases of all patients aged 19–25 years were reviewed in the chosen time-period. 6,989 patients presented for traumatic complaints, of which 200 cases were randomly selected for the comparison group.

### Descriptive data

Most patients (*n* = 1,140, 70.1%) were triaged into category 3 (urgent); only 16.1% of cases (*n* = 262) were labelled highly urgent and 5.7% (*n* = 93) life-threatening. 185 cases (11.4%) overall were treated in our emergency care room for trauma. The most common type of admission was walk-in (*n* = 1,027, 63.2%), followed by admission by ambulance (*n* = 208, 12.8%). Injuries were predominantly caused by sports accidents (*n* = 603, 37.1%), falls (*n* = 204, 12.5%) or violence (*n* = 141, 8.7%). Patients most often presented with injuries to the upper extremities (*n* = 572, 35.2%), followed by injuries to the lower extremities (*n* = 544, 33.5%) and head injuries (*n* = 470, 28.9%). Most patients (86%, *n* = 1,399) suffered multiple injuries (Table 1). 13.2% (*n* = 215) of all patients had to undergo surgery as a form of treatment, 220 patients (13.5%) had to be hospitalised (inpatient) while most patients (*n* = 1,287, 79.2%) could be discharged home (outpatient). 173 patients (10.6%) were hospitalised in our ICU unit. There were two fatal accidents recorded in our data cohorts; they died during inpatient treatment (one due to a fall from height, one due to suicide by a gun). The median cost for the ED stay was 715 Swiss francs (IQR 447–1,229), for the overall inpatient treatment cost 766 Swiss francs (IQR 474–1,481); see Table 1.

### Comparison of sexes

We found that male patients presented to our ED more than twice as often as female patients (*n* = 1,097, 67.5% vs. *n* = 529, 32.5%) (*p* < 0.001). Male patients presented significantly more often during the weekend (*n* = 425, 38.7%) vs. females (*n* = 165, 31.2%) (*p* = 0.003) (Table 1).

In both sexes, sport accidents were the most common mechanism of injury (female *n* = 182, 34.4%, male *n* = 421, 38.4%), yet male patients presented significantly more often with injuries sustained from violence (*n* = 123, 11.2% vs. female: *n* = 18, 3.4%) (*p* < 0.001) (Table 1).

We also found a significant difference between sexes with respect to injured body parts. Female patients predominantly presented with injuries to the lower extremities (*n* = 197, 37.2% vs. male: *n* = 347, 31.6%) (*p* = 0.025) and spine (*n* = 47, 8.9% vs. male: *n* = 53, 4.8%) (*p* = 0.001) while male patients more often suffered injuries to the head (*n* = 335, 30.5% vs. female: *n* = 135, 25.5%) (*p =* 0.037) (Table 1).

The most common injuries in male patients were wounds (*n* = 245, 22.3% vs. female: *n* = 103, 19.5%), Female patients suffered more contusions (*n* = 139, 26.3% vs. male: *n* = 228, 20.8%). Male patients presented significantly more often with fractures (*n* = 215, 19.6%) than female patients (*n* = 58, 11%) (*p* < 0.001). Male patients more often needed surgical treatment than female patients (*n* = 167, 15.2% vs. female: *n* = 48, 9.1% (*p* = 0.001), which consequently led to more inpatient treatment for males (*n* = 165, 15%) than females (*n* = 55, 10.4%) (*p* = 0.1) (Table 1).

Our resource analysis showed that male patients generate significantly more costs for overall hospital stay than female patients (median = 792, [IQR 491–1,598] vs. female: median = 719, [IQR 429–1,429]) (*p* = 0.01). Other resources, such as imaging, ICU stay, length of inpatient treatment stay or ED costs showed no significant differences between the sexes (Table 1).

**Table 1:** Consultation and patient characteristics with sex comparison

Variables	Sex		Odds Ratio		p-value
	Female (*n* = 529)	Male (*n* = 1097)	Adjusted Odds Ratio	(95% CI)	
Age [years]	17 (16–18)	17 (16–18)	1.245	(1.096;1.414)	**0.001**
**Sex**					
Female	529 (100-0)	0 (0.0)	0.000	(Baseline)	
**Day oft the week**					
Monday	73 (13.8)	154 (14.0)	1.000	(Baseline)	
Tuesday	67 (12.7)	139 (12.7)	0.983	(0.657;1.472)	0.935
Wednesday	67 (12.7)	141 (12.9)	0.998	(0.667;1.492)	0.991
Thursday	73 (13.8)	108 (9.8)	0.701	(0.467;1.054)	*0.088*
Friday	84 (15.9)	130 (11.8)	0.734	(0.496;1.085)	0.120
Saturday	68 (12.9)	202 (18.4)	1.408	(0.952;2.082)	*0.086*
Sunday	97 (18.3)	223 (20.3)	1.090	(0.755;1.572)	0.646
**Shift of admission**					
Day (6am to 5 pm)	250 (47.3)	530 (48.3)	1.000	(Baseline)	
Evening (5pm to 10pm)	171 (32.3)	316 (28.8)	0.872	(0.686;1.107)	0.261
Night (10pm to 6 am)	108 (20.4)	251(22.9)	1.096	(0.836;1.438)	0.506
Saturday or Sunday admission	165 (31.2)	425 (38.7)	1.395	(1.119;1.739)	**0.003**
Public and cantonal (Bern) holidays	10 (1.9)	14 (1.3)	0.671	(0.296;1.521)	0.339
**Type of admission**					
Ambulance	65 (12.3)	143 (13.0)	1.000	(Baseline)	
General Practitioner	13 (2.5)	24 (2.2)	0.839	(0.402;1.752)	0.640
External Hospital	16 (3.0)	50 (4.6)	1.420	(0.753;2.680)	0.278
Police	1 (0.2)	2 (0.2)	0.909	(0.081;10.206)	0.938
Air Rescue	25 (4.7)	41 (3.7)	0.745		0.319
Repatriation	1 (0.2)	1 (0.1)	0.455	(0.418;1.328)	0.579
Walk-In	346 (65.4)	681 (62.1)	0.895	(0.028;7.380)	0.469
Internal Referral	1 (0.2)	9 (0.8)	4.091	(0.649;1.233)	*0.186*
Urgent care centre/doctor Other	6 (1.1) 1 (0.2)	12 (1.1) 0 (0.0)	0.909 -	(0.508;32.965) (0.327;2.528)	0.855 -
No Information	54 (10.2)	134 (12.2)	1.128	- (0733;1.736)	0.584
Documented walk-in	346 (65.4)	681 (62.1)	0.866	(0.697;1.075)	*0.193*
**Triage**					
Life-threatening = ESI 1	31 (5.9)	62 (5.7)	1.000	(Baseline)	
High urgent = ESI 2	75 (14.2)	187 (17.0)	1.247	(0.750;2.071)	0.395
Urgent = ESI 3	382 (72.2)	758 (69.1)	0.992	(0.634;1.553)	0.973
Semi-urgent = ESI 4	32(6.0)	67 (6.1)	1.047	(0.573;1.913)	0.882
Non-urgent = ESI 5	2 (0.4)	11 (1.0)	2.750	(0.574;13.180)	0.206
Missing	7 (1.3)	12 (1.1)	0.857	(0.307;2.394)	0.769
Documented high urgency	106 (20.0)	249 (22.7)	1.172	(0.908;1.513)	0.224

### Associations of mechanism of injury and injured body part with treatment procedure

Apart from the greater rate of surgery for males, we found a significant difference between conservative and surgical treatment with respect to mechanisms of injury after motor vehicle accidents (MVA) (surgical (*n* = 30, 14%) vs. conservative treatment (*n* = 102, 7.2%) (*p* = 0.001) and injuries that resulted from violence (surgical (*n* = 27, 12.6%) vs. conservative treatment (*n* = 114, 8.1%, *p* = 0.03) (Supplement 1). Injuries sustained from sport accidents were more likely to be treated conservatively (*n* = 543, 38.5%) than surgically (*n* = 60, 27.9%) (*p* = 0.003). We also found that higher surgery rates were significantly associated with injuries to the upper extremities (OR 1.6 [1.2–2.2], *p* = 0.001) and multiple body parts (OR 1.7 [1.0–2.7], *p* = 0.036) (Supplement 1). The only significant factor for a greater rate of inpatient treatment was self-harm (*n* = 8, 33.3% vs. non-hospitalised: *n* = 41, 2.6%, *p* < 0.001).

### Multivariable analysis

**Fig. 2:**
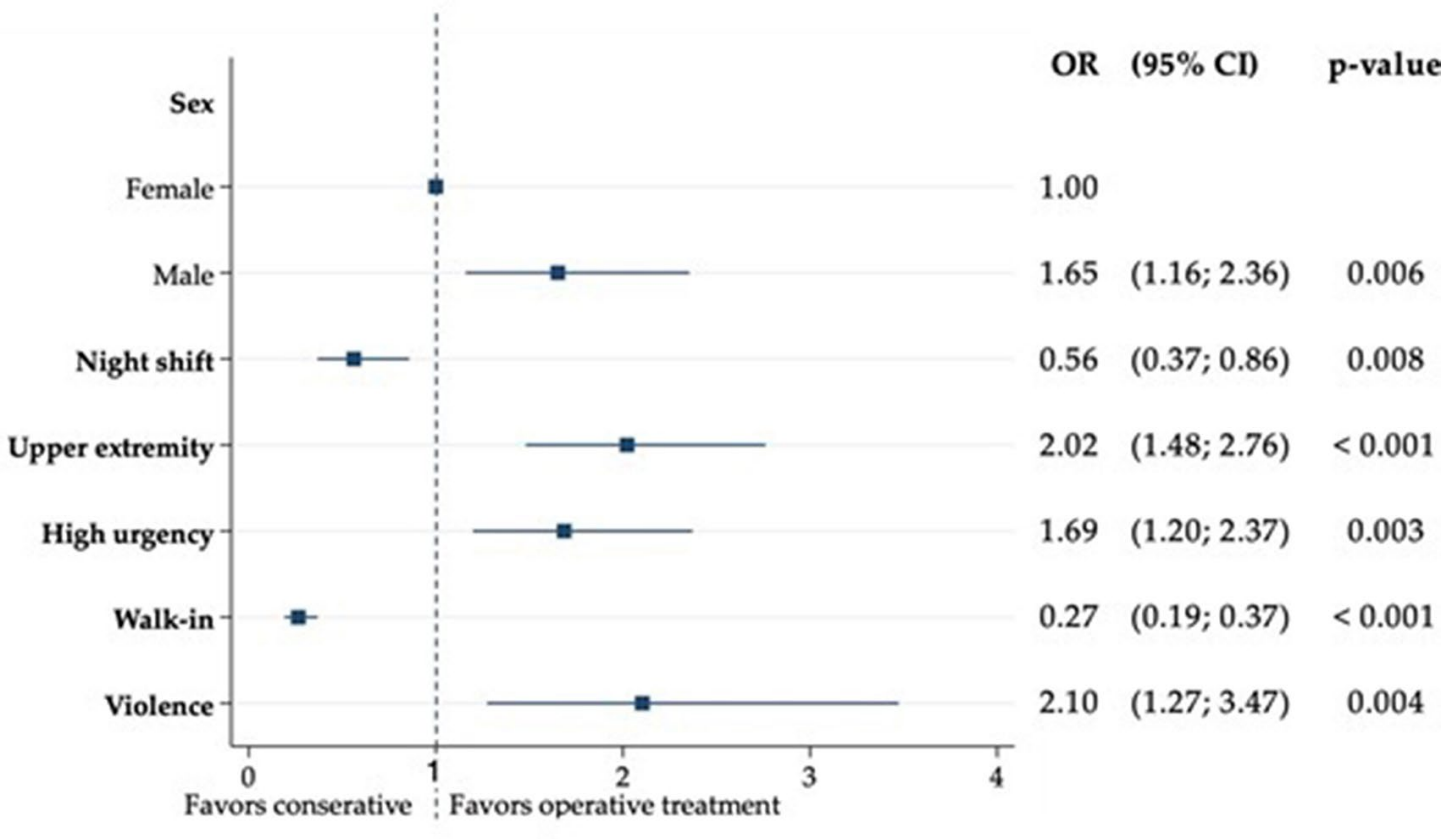
Multivariable analysis of factors favouring greater rates of surgical treatment in adolescents of 16–18 years old

Multivariable analysis was used to determine factors that favour surgical treatment. Variables that showed at least a weak association in univariable analysis (*p* < 0.20) (Supplement 1) were used in stepwise backward logistic regression [17, 18]. Demographic characteristics that showed significant association with higher operation rates were male sex (OR 1.7 [95% CI: 1.2–2.4], *p* = 0.006), high urgency triage group (OR 1.7 [95% CI: 1.2–2.4], *p* = 0.003), upper extremity injuries (OR 2.0 [95% CI: 1.5–2.8], *p* < 0.001), as well as injuries by violence (OR 2.1 [95% CI: 1.3–3.5] *p* = 0.004). Presentation at night (OR 0.6 [95% CI: 0.4–0.9], *p* = 0.008) or as walk-in patients was significantly protective for surgical treatment (OR 0.3 [95% CI: 0.2–0.4], *p* < 0.001) (Fig. 2).

### Case group vs. comparison group (young adults 19–25 years old)

**Fig. 3:**
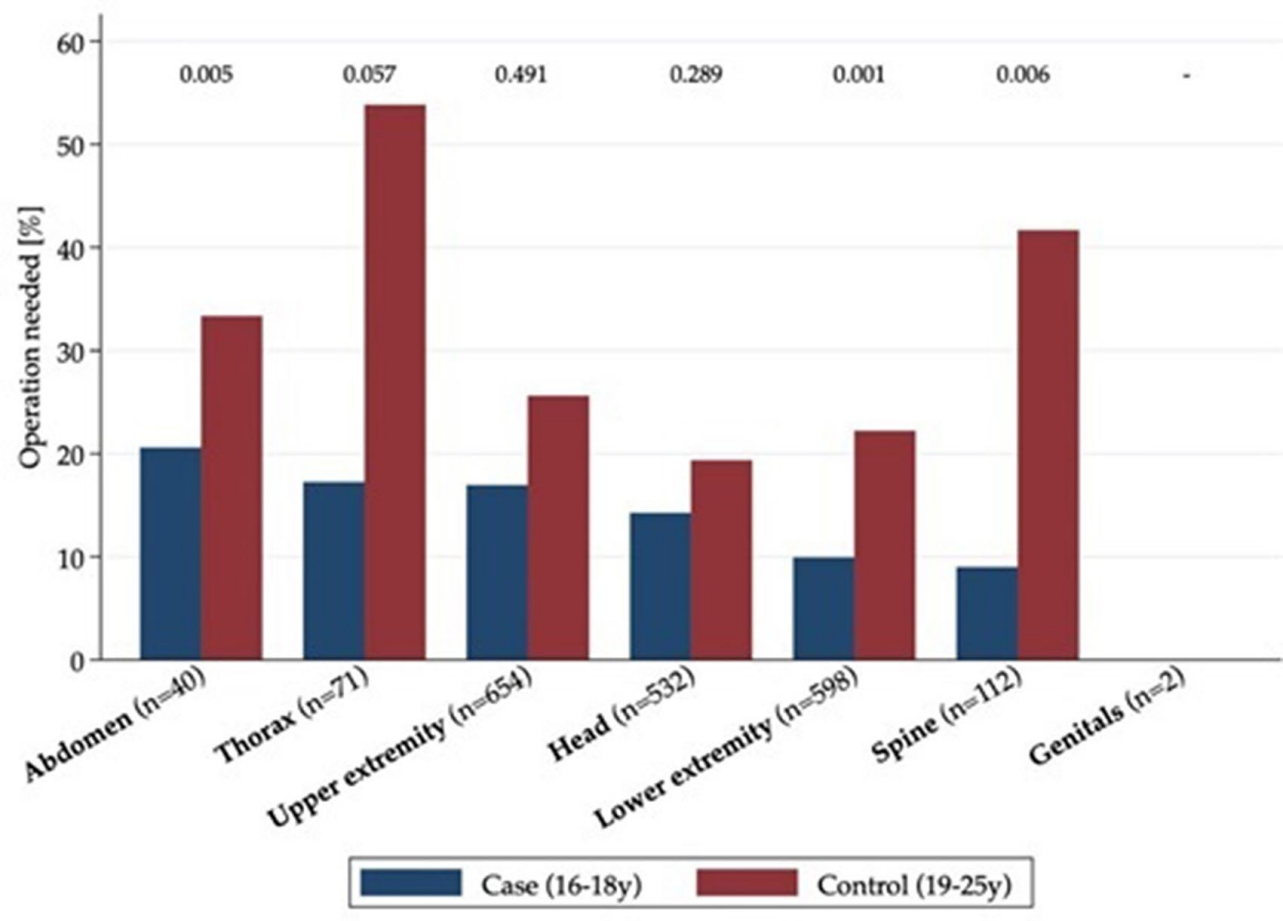
Comparison of case and comparison groups for surgical treatment by injured body part

Overall, presentation characteristics were similar between 19 and 25 year old and 16 to 18 year old patients. In the comparison group, 36.8% of all patients were female. Most patients in this group presented as walk-in cases (59.6%), most frequently on Sundays (21.2%). As in the case group, the majority of all patients were triaged into category 3 (urgent) (74.1%) and could be discharged home (79.2%). A significant difference was found between cases and controls in regard to sport accidents. 37.1% of adolescents (*n* = 603) presented after sport accidents and 21.2% of the adults (*n* = 41). Comparison group patients more often presented with wounds (32.1%, *n* = 62) (Supplement 2). Both groups most often presented at our ED with injuries to the upper extremities (*p* = 0.046) and lower extremities (*p* = 0.126). Older patients were significantly more often injured in the thorax (*n* = 13, 6.7% vs. cases: *n* = 58, 3.6%) (*p* = 0.035). Surgical treatment was significantly more often necessary in the comparison group (*n* = 39, 20.2% vs. cases: *n* = 215, 13.2%) (*p* = 0.09), and this was significant for trauma to the spine (*p* = 0.006), abdomen (*p* = 0.005) and lower extremities (*p* = 0.001) (Fig. 3). The greater rate of surgery was also reflected in the inpatient treatment costs (median cases: 766 Swiss francs [IQR 474–1,229], median controls: 792 Swiss francs [IQR 530–1,511]) (*p* = 0.012). The mortality rate was ten times higher in the comparison group (1%, *n* = 2) than in the case group (0.1%, *n* = 2) (*p* = 0.33) (Supplement 2). Multivariable analysis for the comparison group using the identified confounders as in the case group showed significantly greater association with surgical treatment for male patients (*p* = 0.018), and injury to the upper extremities (*p* = 0.016) and less association with surgical treatment for presentation at night (*p* = 0.042) (Supplement 3). Associations with surgical treatment for high urgency and walk-in cases as well as injuries obtained by violence did not show a significant association. The magnitude and direction of the association was similar to that in the case group (Supplement 3).

## Discussion

### Key results

Between January 2013 and July 2017, 1,626 adolescents aged 16–18 presented to the adult ED at the University Hospital of Bern for trauma—most commonly due to sport accidents. Overall, male adolescents were more likely to present to our ED than females. Most patients presented with multiple injuries, most commonly to the upper extremities and caused by contusions or wounds. Surgical and inpatient treatment rates were similar, with 13.2% and 13.5%. A high proportion of patients had to be treated in our ICU unit (10.6%). In-hospital mortality was low in this patient collective (0.1%). Male patients more often presented with wounds and fractures and were more likely to be surgically treated and hospitalised.

Multivariable analysis showed significant associations for male sex, high urgency triage, upper extremity injuries and injuries caused by violence, while presentation as walk-in patients and at night were protective for surgical treatment. Overall, 19–25 year old patients tend to present to our ED with more severe trauma, and this led to a higher rate of surgery as well as higher mortality.

### Comparison with other studies

A great deal of adolescent medical care occurs in the ED [19]. Several ED studies, including our initial study, found that injuries were the most common cause of adolescent presentations to the ED [11, 20–22]. Furthermore, studies have shown that in the age group of adolescents, males (52–60%) present to the ED more often than females [6, 7, 20, 21, 23, 24]. For the subgroup of presentations due to trauma, this difference is even greater. These findings correlate well with our data, which showed that only 32.5% of presentations are female.

We found further sex-specific differences in the mechanism of trauma and affected body part. Our data suggests that male patients more often suffered fractures and that the injuries were more often caused by violence. A Swedish study showed that most presentations to the ED of male patients between 15 and 17 years old were associated with violence [25]. Female patients on the other hand more often suffered injuries to the lower extremities and less frequently required inpatient treatment [25]. Our data also showed greater rates of surgery and inpatient treatment for male patients, especially when combined with trauma through violence.

A large US study examined paediatric emergency presentations for trauma between 2006 and 2012 and found that the most common reasons for presentation were wounds, contusions, sprains and strains, and fractures [24]. These findings overlap with our results as well. The number of ED visits in this age group has remained roughly constant in Switzerland between 2013 and 2017, while in this US study the number of presentations of 15–19-year-olds has slightly decreased [24].

In comparison to our comparison group of 19–25 years old patients, adolescents more often presented to our ED after sports accidents. This might be due to the fact that younger patients generally still attend mandatory physical education classes [26].

Furthermore, mortality is strikingly high in the comparison group, This contrasts with the findings of an American study from 2018, in which the younger population has a higher mortality rate in the ED, although this study compares paediatric (0-14yo) with adult (15-64yo) patients [27]. Moreover, a greater mortality was shown in victims of violence than in non-violent trauma [28]. These results were not reproduced in our study and therefore do not explain our findings.

A Germany-wide study from 2014 examined the occurrence of accidents in children and adolescents in 2011. The authors were able to show that 13.8% of the patients had to be treated as inpatients due to an accident. Our analysis showed that 13.5% of patients needed inpatient treatment. In absolute terms, injuries to the head were by far the most frequent reason for inpatient treatment, followed by injuries to the upper and lower extremities [6]. We found that injuries to the upper extremities were associated with greater rates of surgical treatment and consequently of inpatient treatment.

Adolescents prefer to present as walk-in patients [21] and do so conspicuously often during the weekends [20]; this has also been observed in other studies. The most frequent triages in a study conducted in an Australian ED were—similar to our patients’ collective—semi-urgent (4/5) and non-urgent (5/5) [20]. Our data showed that there was a protective association with surgical treatment for walk-in patients.

A UK study from 2021 suggests that children-specific trauma care centres achieve a significantly lower mortality rate in trauma-related presentations of adolescents patients [29]—although the specific factors for this lower mortality are still unknown. However, the adolescent patient group was defined as 11–25 year old patients, which differs greatly from our adolescent patient collective. Our cases group only showed a few statistically significant differences to the comparison group of young adults, though the mortality rate was found to be lower in the case group. Overall, the mortality rate in our patient collective was comparatively low (0.1% in the adolescent group) compared to the mortality rate in the UK study (2.5–4.9% for different trauma centres) [29].

### Interpretation

Only a few studies have concentrated on the specific age group of 16–18 year olds. The first publication of the ED of the Inselspital Bern was able to show significant differences in presentations due to trauma and psychiatric emergencies compared to the adult population [11]. Psychiatric emergencies have already been analysed in detail in a follow-up paper [12]; this publication now addresses trauma-related presentations.

The main objective of this study was, on the one hand, to provide a more detailed analysis of the specific subset of trauma-related adolescent admissions to adult EDs, with comparisons between male and female patients as well as between adolescents (case group) and young adults (comparison group)—in terms of trauma mechanisms, body parts affected, therapeutic consequences, as well as inpatient treatment, ICU stay and costs. On the other hand, we conducted a multivariable analysis to determine predictive factors for surgical treatment.

Although adolescents should not be considered and treated as adults, this is the case in many large hospitals and their EDs in Switzerland; therefore, this topic has at least national, if not international, relevance.

The transitional phase from childhood to adulthood and its characteristics have been very poorly researched. It has frequently been found that trauma assessment and treatment of children differ from that of adults [7, 8, 30]. Therefore, the attending medical and nursing staff should ideally have expertise in both areas.

To the best of our knowledge, of the few studies that examined adolescent presentations to the ED and their reasons, only a minority focused on trauma and even fewer on the mechanisms of injury or the treatments. For adolescents, our study showed that 20% of patients with abdominal trauma need surgical intervention. Blunt abdominal trauma is the third most common cause of death from paediatric trauma but is the most common unrecognised fatal injury. FAST (focused assessment with sonography in trauma) has a low sensitivity (66%) in the haemodynamically stable paediatric trauma patient [7] but is still useful, available and cheap. Therefore, the indication for exploratory laparoscopy should be discussed at an early stage.

Various prevention programmes already exist throughout Switzerland and are an important factor in the prevention of injuries [31–34]. On the basis of our new findings, these could be further targeted to the affected subgroup. We feel that injury prevention for the adolescent patient group should focus on sport-related accidents (presumably targeted at mandatory physical education classes in schools), as well as prevention of violence.

For process and resource optimisation, we constantly collect data and conduct analysis on our emergency ward [12, 35–38]. This should and will be maintained in order to achieve the best possible triaging, diagnosis and therapy of the particularly vulnerable subgroup of adolescents. We hope to achieve a standardised optimal process flow on our ED, especially for presentations due to trauma.

### Strength and limitations

A key limitation of the previously conducted review study was that it was based on data collected at triage. This weakness was addressed in this study and replaced with discharge data as well as inpatient treatment data, and this is a clear strength of our study. The study design is retrospective, with data from a single centre and using routine hospital data and this could potentially lead to information bias. Additionally, exclusion of cases with negative GC is known to increase the risk of selection bias [39–41].

Unfortunately, vital parameters as well as parameters for risk scores (e.g. Injury Severity Score) could not be included into the data analysis—due to missing data collection. For more detailed analysis and better prediction of outcome from injury severity, more extensive data research and collection would be necessary.

In addition, on the basis of the data we collected, it was not possible to conclusively assess the cause of the significant differences in the rates of surgery. One possible explanation could be the more liberal indication for admission to the ED in adolescents, as fewer serious injuries lead to lower rates of surgery in adolescent patients.

In the city of Bern and the adjacent rural areas, there are a total of seven other emergency wards—Hirslanden Klinik (Salemspital, Klinik Beausite, Klinik Permanence), Tiefenauspital Inselgruppe, and Lindenhofgruppe (Lindenhofspital, Klinik Sonnenhof, City Notfall)–, but the ED of the Inselspital University Hospital is by far the largest and most frequently visited. This could lead to a selection bias towards more severe cases. To date, we do not have any studies on adolescent emergency presentation from the other hospitals. In order to complete our data and increase the significance, collected data from all EDs should be obtained. Additionally, this study is limited to a single centre in Switzerland, which unfortunately makes the findings more difficult to generalise.

## Conclusions

This study provides a summary of presentations to the ED due to trauma by adolescents aged 16–18 years and reveals significant sex- and age-specific differences. Additionally, it shows specific predictive factors for higher rates of surgery amongst adolescent patients. Male adolescents account for the majority of emergency visits. Overall, we found significant associations with surgical treatment for male sex, high urgency triage, upper extremity injuries and injuries caused by violence, while presentation at night and as walk-in patients showed protective association. We recommend precise diagnostic and early surgical consultation, especially for patients with abdominal trauma. Preventive measures and recommendations should preferably focus on sport activities and violence.

### Electronic supplementary material

Below is the link to the electronic supplementary material.

**Supplement Table 1:** Comparison of treatment operation in the validation set using logistic regression analysis.

**Supplement Table 2:** Comparison to comparison group (young adults 19-25 years old) in the validation set using logistic regression analysis.

**Supplement Table 3:** Multivariable analysis for the comparison group

## Data Availability

The data underlying this article will be shared on reasonable request to the corresponding author.
