# Supplementary material for: Emergency department utilisation and treatment for trauma-related presentations of adolescents aged 16–18: a retrospective cross-sectional study
Source: BMC Emerg Med. 2024 Feb 27;24:33. doi: 10.1186/s12873-024-00945-8 (PMC10900568; doi:10.1186/s12873-024-00945-8)
Supplement: Supplementary file 1 — Supplement Table 1: Comparison of treatment operation in the validation set using logistic regression analysis. [file 12873_2024_945_MOESM1_ESM.docx]

| **Variables** | **Treatment operation** |  |  |  |  | **Odds Ratio** |  |  |
| --- | --- | --- | --- | --- | --- | --- | --- | --- |
|  | **N** | **(n=1411)** | **Yes** | **(n=215)** | **p-value** | **Adjusted Odds Ratio** | **(95% CI)** | **p-value*** |
| **Age [years]** | 17 | (16 - 18) | 17 | (17 - 18) | 0.042 | 1.205 | (1.008;1.440) | **0.040** |
| **Sex** |  |  |  |  |  |  |  |  |
| Female | 481 | (34.1) | 48 | (22.3) |  | 1.000 | (Baseline) |  |
| Male | 930 | (65.9) | 167 | (77.7) | 0.001 | 1.799 | (1.282;2.526) | **0.001** |
| **Day of week** |  |  |  |  |  |  |  |  |
| Monday | 202 | (14.3) | 25 | (11.6) |  | 1.000 | (Baseline) |  |
| Tuesday | 184 | (13.0) | 22 | (10.2) |  | 0.966 | (0.527;1.772) | 0.911 |
| Wednesday | 177 | (12.5) | 31 | (14.4) |  | 1.415 | (0.805;2.488) | 0.228 |
| Thursday | 155 | (11.0) | 26 | (12.1) |  | 1.355 | (0.753;2.439) | 0.310 |
| Friday | 190 | (13.5) | 24 | (11.2) |  | 1.021 | (0.563;1.849) | 0.946 |
| Saturday | 233 | (16.5) | 37 | (17.2) |  | 1.283 | (0.747;2.205) | 0.367 |
| Sunday | 270 | (19.1) | 50 | (23.3) | 0.511 | 1.496 | (0.895;2.501) | *0.124* |
| **Shift of admission** |  |  |  |  |  |  |  |  |
| Day (6am to 5 pm) | 665 | (47.1) | 115 | (53.5) |  | 1.000 | (Baseline) |  |
| Evening (5pm to 10pm) | 421 | (29.8) | 66 | (30.7) |  | 0.907 | (0.654;1.256) | 0.556 |
| Night (10pm to 6 am) | 325 | (23.0) | 34 | (15.8) | 0.049 | 0.605 | (0.404;0.907) | **0.015** |
| **Saturday or Sunday admission** | 503 | (35.6) | 87 | (40.5) | 0.171 | 1.227 | (0.915;1.645) | *0.172* |
| **Public and cantonal (Bern) holidays** | 20 | (1.4) | 4 | (1.9) | 0.616 | 1.318 | (0.446;3.895) | 0.617 |
| **Documented walk-in** | 956 | (67.8) | 71 | (33.0) | <0.001 | 0.235 | (0.173;0.318) | **<0.001** |
| **Documented high urgency** | 275 | (19.5) | 80 | (37.2) | <0.001 | 2.448 | (1.802;3.325) | **<0.001** |
| **Treatment emergency trauma room** | 137 | (9.7) | 48 | (22.3) | <0.001 | 2.673 | (1.853;3.855) | **<0.001** |
| **Injury mechanism** |  |  |  |  |  |  |  |  |
| Fall | 181 | (12.8) | 23 | (10.7) |  | 1.000 | (Baseline) |  |
| Bicycle accident | 66 | (4.7) | 14 | (6.5) |  | 1.669 | (0.811;3.435) | *0.164* |
| Motor vehicle accident | 102 | (7.2) | 30 | (14.0) |  | 2.315 | (1.277;4.196) | **0.006** |
| Violence | 114 | (8.1) | 27 | (12.6) |  | 1.864 | (1.019;3.408) | **0.043** |
| Selfharm | 43 | (3.0) | 6 | (2.8) |  | 1.098 | (0.421;2.862) | 0.848 |
| Injuries from heat / cold | 25 | (1.8) | 1 | (0.5) |  | 0.315 | (0.041;2.434) | 0.268 |
| Sport accident | 543 | (38.5) | 60 | (27.9) |  | 0.870 | (0.523;1.447) | 0.591 |
| No information/Other | 337 | (23.9) | 54 | (25.1) | 0.001 | 1.261 | (0.749;2.122) | 0.382 |
| **Category diagnosis** |  |  |  |  |  |  |  |  |
| Fracture | 158 | (11.2) | 115 | (53.5) |  | 1.000 | (Baseline) |  |
| Contusion | 364 | (25.8) | 3 | (1.4) |  | 0.011 | (0.004;0.036) | **<0.001** |
| Sprain/distorsion | 288 | (20.4) | 8 | (3.7) |  | 0.038 | (0.018;0.080) | **<0.001** |
| Concussion | 146 | (10.3) | 5 | (2.3) |  | 0.047 | (0.019;0.118) | **<0.001** |
| Wound | 298 | (21.1) | 50 | (23.3) |  | 0.231 | (0.157;0.338) | **<0.001** |
| No information | 122 | (8.6) | 13 | (6.0) |  | 0.146 | (0.079;0.272) | **<0.001** |
| Multiple trauma | 35 | (2.5) | 21 | (9.8) | <0.001 | 0.824 | (0.456;1.490) | 0.522 |
| **Injured Body Part** |  |  |  |  |  |  |  |  |
| Head | 403 | (28.6) | 67 | (31.2) | 0.433 | 1.132 | (0.830;1.545) | 0.433 |
| Upper extremity | 475 | (33.7) | 97 | (45.1) | 0.001 | 1.620 | (1.212;2.166) | **0.001** |
| Lower extremity | 490 | (34.7) | 54 | (25.1) | 0.005 | 0.630 | (0.455;0.874) | **0.006** |
| Thorax | 48 | (3.4) | 10 | (4.7) | 0.358 | 1.385 | (0.690;2.781) | 0.360 |
| Abdomen | 27 | (1.9) | 7 | (3.3) | 0.200 | 1.725 | (0.742;4.012) | 0.205 |
| Spine | 91 | (6.4) | 9 | (4.2) | 0.198 | 0.634 | (0.315;1.277) | 0.202 |
| Genitals | 2 | (0.1) | 0 | (0.0) | 0.581 | - |  |  |
| Multiple body part | 1204 | (85.3) | 195 | (90.7) | 0.034 | 1.676 | (1.034;2.718) | **0.036** |
| **Imaging** |  |  |  |  |  |  |  |  |
| X-ray performed | 881 | (62.4) | 148 | (68.8) | 0.070 | 1.329 | (0.977;1.808) | *0.070* |
| Sonography performed | 188 | (13.3) | 32 | (14.9) | 0.533 | 1.138 | (0.758;1.707) | 0.534 |
| CT performed | 338 | (24.0) | 84 | (39.1) | <0.001 | 2.036 | (1.508;2.748) | **<0.001** |
| MRI performed | 85 | (6.0) | 8 | (3.7) | 0.176 | 0.603 | (0.288;1.263) | *0.180* |
| **Way of discharge** |  |  |  |  |  |  |  |  |
| At home | 1231 | (87.2) | 56 | (26.0) |  | 1.000 | (Baseline) |  |
| Hospital admission | 72 | (5.1) | 148 | (68.8) |  | 45.186 | (30.635;66.646) | **<0.001** |
| Transfer to external hospital | 20 | (1.4) | 4 | (1.9) |  | 4.396 | (1.454;13.293) | **0.009** |
| No information | 88 | (6.2) | 7 | (3.3) | <0.001 | 1.749 | (0.774;3.950) | *0.179* |
| **Treatment operation** | 0 | (0.0) | 215 | (100.0) | <0.001 | - |  |  |
| **ICU stay** | 132 | (9.4) | 41 | (19.1) | <0.001 | 2.283 | (1.554;3.354) | **<0.001** |
| **LOS hospital [days]** | 0.1 | (0.1 - 0.2) | 2.1 | (0.2 - 5.1) | <0.001 | 0.039 | (0.034;0.043) | **<0.001** |
| **In-hospital death** | 2 | (0.1) | 0 | (0.0) | 0.581 | - |  |  |
| **Total cost ED [Swissfrancs]** | 666 | (433 - 1144) | 1125 | (658 - 2293) | <0.001 | 1.000 | (1.000;1.001) | **<0.001** |
| **Total cost hospital [Swissfrancs]** | 689 | (444 - 1184) | 7417 | (1456 - 17159) | <0.001 | 1.000 | (1.000;1.000) | **<0.001** |

Categorical variables are shown with number (%) in each category, p-values obtained by Chi-squared test. Continous variables are described with median (IQR), p-values obtained by Wilcoxon rank sum test.

* bold = p-value beneath statistical significance level (< 0.05). italic = p-value at least weak association (< 0.2)

**Supplement table 1:** Comparison of treatment operation in the validation set using logistic regression analysis.
