# Supplementary material for: Emergency department utilisation and treatment for trauma-related presentations of adolescents aged 16–18: a retrospective cross-sectional study
Source: BMC Emerg Med. 2024 Feb 27;24:33. doi: 10.1186/s12873-024-00945-8 (PMC10900568; doi:10.1186/s12873-024-00945-8)
Supplement: Supplementary file 2 — Supplement Table 2: Comparison to comparison group (young adults 19-25 years old) in the validation set using logistic regression analysis. [file 12873_2024_945_MOESM2_ESM.docx]

| **Variables** |  | **Total** |  | **Control group** |  |  |  |  | **Odds Ratio** |  | |  | |
| --- | --- | --- | --- | --- | --- | --- | --- | --- | --- | --- | --- | --- | --- |
|  | **N** | **Total** | **(n=1819)** | **Case (16-18y)** | **(n=1626)** | **Control (19-25y)** | **(n=193)** | **p-value** | **Adjusted Odds Ratio** | **(95% CI)** | **p-value*** | |  |
| **Age [years]** | 1819 | 17 | (16 - 18) | 17 | (16 - 18) | 22 | (20 - 24) | <0.001 | 0.138 | (0.134;0.142) | | **<0.001** | |
| **Sex** | 1819 |  |  |  |  |  |  |  |  |  | |  | |
| Female |  | 600 | (33.0) | 529 | (32.5) | 71 | (36.8) |  | 1.000 | (Baseline) | |  | |
| Male |  | 1219 | (67.0) | 1097 | (67.5) | 122 | (63.2) | 0.235 | 0.829 | (0.607;1.130) | | 0.235 | |
| **Day of week** | 1819 |  |  |  |  |  |  |  |  |  | |  | |
| Monday |  | 258 | (14.2) | 227 | (14.0) | 31 | (16.1) |  | 1.000 | (Baseline) | |  | |
| Tuesday |  | 229 | (12.6) | 206 | (12.7) | 23 | (11.9) |  | 0.818 | (0.462;1.448) | | 0.490 | |
| Wednesday |  | 231 | (12.7) | 208 | (12.8) | 23 | (11.9) |  | 0.810 | (0.457;1.434) | | 0.469 | |
| Thursday |  | 208 | (11.4) | 181 | (11.1) | 27 | (14.0) |  | 1.092 | (0.629;1.896) | | 0.754 | |
| Friday |  | 234 | (12.9) | 214 | (13.2) | 20 | (10.4) |  | 0.684 | (0.378;1.237) | | 0.209 | |
| Saturday |  | 298 | (16.4) | 270 | (16.6) | 28 | (14.5) |  | 0.759 | (0.442;1.304) | | 0.318 | |
| Sunday |  | 361 | (19.8) | 320 | (19.7) | 41 | (21.2) | 0.720 | 0.938 | (0.571;1.541) | | 0.801 | |
| **Shift of admission** | 1819 |  |  |  |  |  |  |  |  |  | |  | |
| Day (6am to 5 pm) |  | 876 | (48.2) | 780 | (48.0) | 96 | (49.7) |  | 1.000 | (Baseline) | |  | |
| Evening (5pm to 10pm) |  | 539 | (29.6) | 487 | (30.0) | 52 | (26.9) |  | 0.868 | (0.608;1.238) | | 0.434 | |
| Night (10pm to 6 am) |  | 404 | (22.2) | 359 | (22.1) | 45 | (23.3) | 0.685 | 1.018 | (0.700;1.483) | | 0.924 | |
| **Saturday or Sunday admission** | 1819 | 659 | (36.2) | 590 | (36.3) | 69 | (35.8) | 0.884 | 0.977 | (0.716;1.334) | | 0.884 | |
| **Public and cantonal (Bern) holidays** | 1819 | 26 | (1.4) | 24 | (1.5) | 2 | (1.0) | 0.627 | 0.699 | (0.164;2.981) | | 0.628 | |
| **Type of admission** | 1819 |  |  |  |  |  |  |  |  |  | |  | |
| Ambulance |  | 229 | (12.6) | 208 | (12.8) | 21 | (10.9) |  | 1.000 | (Baseline) | |  | |
| General Practitioner |  | 42 | (2.3) | 37 | (2.3) | 5 | (2.6) |  | 1.338 | (0.475;3.772) | | 0.581 | |
| External Hospital |  | 74 | (4.1) | 66 | (4.1) | 8 | (4.1) |  | 1.201 | (0.508;2.837) | | 0.677 | |
| Police |  | 4 | (0.2) | 3 | (0.2) | 1 | (0.5) |  | 3.302 | (0.329;33.170) | | 0.310 | |
| Air Rescue |  | 74 | (4.1) | 66 | (4.1) | 8 | (4.1) |  | 1.201 | (0.508;2.837) | | 0.677 | |
| Repatriation |  | 2 | (0.1) | 2 | (0.1) | 0 | (0.0) |  | - |  | |  | |
| Walk-In |  | 1142 | (62.8) | 1027 | (63.2) | 115 | (59.6) |  | 1.109 | (0.681;1.808) | | 0.678 | |
| Internal Referral |  | 11 | (0.6) | 10 | (0.6) | 1 | (0.5) |  | 0.990 | (0.121;8.121) | | 0.993 | |
| Urgent care centre/doctor |  | 23 | (1.3) | 18 | (1.1) | 5 | (2.6) |  | 2.751 | (0.927;8.164) | | *0.068* | |
| Other |  | 1 | (0.1) | 1 | (0.1) | 0 | (0.0) |  | - |  | |  | |
| No Information |  | 217 | (11.9) | 188 | (11.6) | 29 | (15.0) | 0.732 | 1.528 | (0.843;2.771) | | *0.163* | |
| **Triage** | 1819 |  |  |  |  |  |  |  |  |  | |  | |
| Life-threatening = ESI 1 |  | 108 | (5.9) | 93 | (5.7) | 15 | (7.8) |  | 1.000 | (Baseline) | |  | |
| High urgent = ESI 2 |  | 284 | (15.6) | 262 | (16.1) | 22 | (11.4) |  | 0.521 | (0.259;1.046) | | *0.067* | |
| Urgent = ESI 3 |  | 1283 | (70.5) | 1140 | (70.1) | 143 | (74.1) |  | 0.778 | (0.439;1.379) | | 0.389 | |
| Semi-urgent = ESI 4 |  | 107 | (5.9) | 99 | (6.1) | 8 | (4.1) |  | 0.501 | (0.203;1.237) | | *0.134* | |
| Non-urgent = ESI 5 |  | 16 | (0.9) | 13 | (0.8) | 3 | (1.6) |  | 1.431 | (0.364;5.623) | | 0.608 | |
| Missing |  | 21 | (1.2) | 19 | (1.2) | 2 | (1.0) | 0.276 | 0.653 | (0.138;3.093) | | 0.591 | |
| **Treatment emergency trauma room** | 1819 | 204 | (11.2) | 185 | (11.4) | 19 | (9.8) | 0.523 | 0.851 | (0.517;1.399) | | 0.524 | |
| **Injury mechanism** | 1819 |  |  |  |  |  |  |  |  |  | |  | |
| Fall |  | 237 | (13.0) | 204 | (12.5) | 33 | (17.1) |  | 1.000 | (Baseline) | |  | |
| Bicycle accident |  | 91 | (5.0) | 80 | (4.9) | 11 | (5.7) |  | 0.850 | (0.410;1.763) | | 0.662 | |
| Motor vehicle accident |  | 146 | (8.0) | 132 | (8.1) | 14 | (7.3) |  | 0.656 | (0.338;1.272) | | 0.212 | |
| Violence |  | 158 | (8.7) | 141 | (8.7) | 17 | (8.8) |  | 0.745 | (0.400;1.390) | | 0.355 | |
| Selfharm |  | 61 | (3.4) | 49 | (3.0) | 12 | (6.2) |  | 1.514 | (0.729;3.143) | | 0.266 | |
| Injuries from heat / cold |  | 28 | (1.5) | 26 | (1.6) | 2 | (1.0) |  | 0.476 | (0.108;2.098) | | 0.326 | |
| Sport accident |  | 644 | (35.4) | 603 | (37.1) | 41 | (21.2) |  | 0.420 | (0.259;0.683) | | **<0.001** | |
| Laceration/cut |  | 1 | (0.1) | 0 | (0.0) | 1 | (0.5) |  | - |  | |  | |
| No information/Other |  | 453 | (24.9) | 391 | (24.0) | 62 | (32.1) | <0.001 | 0.980 | (0.622;1.545) | | 0.931 | |
| **Category diagnosis** | 1819 |  |  |  |  |  |  |  |  |  | |  | |
| Fracture |  | 317 | (17.4) | 273 | (16.8) | 44 | (22.8) |  | 1.000 | (Baseline) | |  | |
| Contusion |  | 395 | (21.7) | 367 | (22.6) | 28 | (14.5) |  | 0.473 | (0.287;0.780) | | **0.003** | |
| Sprain/distorsion |  | 330 | (18.1) | 296 | (18.2) | 34 | (17.6) |  | 0.713 | (0.442;1.148) | | *0.164* | |
| Concussion |  | 167 | (9.2) | 151 | (9.3) | 16 | (8.3) |  | 0.657 | (0.359;1.205) | | *0.175* | |
| Wound |  | 410 | (22.5) | 348 | (21.4) | 62 | (32.1) |  | 1.105 | (0.728;1.678) | | 0.638 | |
| No information |  | 136 | (7.5) | 135 | (8.3) | 1 | (0.5) |  | 0.046 | (0.006;0.337) | | **0.002** | |
| Multiple trauma |  | 64 | (3.5) | 56 | (3.4) | 8 | (4.1) | <0.001 | 0.886 | (0.396;1.985) | | 0.769 | |
| **Injured Body Part** |  |  |  |  |  |  |  |  |  |  | |  | |
| Head | 1819 | 532 | (29.2) | 470 | (28.9) | 62 | (32.1) | 0.353 | 1.164 | (0.845;1.604) | | 0.353 | |
| Upper extremity | 1819 | 654 | (36.0) | 572 | (35.2) | 82 | (42.5) | 0.045 | 1.361 | (1.005;1.843) | | **0.046** | |
| Lower extremity | 1819 | 598 | (32.9) | 544 | (33.5) | 54 | (28.0) | 0.126 | 0.773 | (0.555;1.076) | | *0.126* | |
| Thorax | 1819 | 71 | (3.9) | 58 | (3.6) | 13 | (6.7) | 0.032 | 1.952 | (1.049;3.633) | | **0.035** | |
| Abdomen | 1819 | 40 | (2.2) | 34 | (2.1) | 6 | (3.1) | 0.362 | 1.502 | (0.623;3.626) | | 0.365 | |
| Spine | 1819 | 112 | (6.2) | 100 | (6.2) | 12 | (6.2) | 0.971 | 1.012 | (0.545;1.877) | | 0.971 | |
| Genitals | 1819 | 2 | (0.1) | 2 | (0.1) | 0 | (0.0) | 0.626 | - |  | |  | |
| Multiple body part | 1819 | 1563 | (85.9) | 1399 | (86.0) | 164 | (85.0) | 0.687 | 0.918 | (0.604;1.395) | | 0.687 | |
| **Imaging** |  |  |  |  |  |  |  |  |  |  | |  | |
| X-ray performed | 1819 | 1139 | (62.6) | 1029 | (63.3) | 110 | (57.0) | 0.088 | 0.769 | (0.568;1.040) | | *0.088* | |
| Sonography performed | 1819 | 238 | (13.1) | 220 | (13.5) | 18 | (9.3) | 0.102 | 0.657 | (0.397;1.090) | | *0.104* | |
| CT performed | 1819 | 466 | (25.6) | 422 | (26.0) | 44 | (22.8) | 0.342 | 0.843 | (0.591;1.200) | | 0.343 | |
| MRI performed | 1819 | 101 | (5.6) | 93 | (5.7) | 8 | (4.1) | 0.366 | 0.713 | (0.341;1.491) | | 0.369 | |
| **Way of discharge** | 1819 |  |  |  |  |  |  |  |  |  | |  | |
| At home |  | 1435 | (78.9) | 1287 | (79.2) | 148 | (76.7) |  | 1.000 | (Baseline) | |  | |
| Hospital admission |  | 252 | (13.9) | 220 | (13.5) | 32 | (16.6) |  | 1.265 | (0.841;1.902) | | 0.259 | |
| Transfer to external hospital |  | 28 | (1.5) | 24 | (1.5) | 4 | (2.1) |  | 1.449 | (0.496;4.234) | | 0.497 | |
| ED death |  | 1 | (0.1) | 0 | (0.0) | 1 | (0.5) |  | - |  | |  | |
| No information |  | 103 | (5.7) | 95 | (5.8) | 8 | (4.1) | 0.027 | 0.732 | (0.349;1.537) | | 0.410 | |
| **Treatment operation** | 1819 | 254 | (14.0) | 215 | (13.2) | 39 | (20.2) | 0.008 | 1.662 | (1.137;2.429) | | **0.009** | |
| **ICU stay** | 1819 | 192 | (10.6) | 173 | (10.6) | 19 | (9.8) | 0.734 | 0.917 | (0.557;1.511) | | 0.734 | |
| **LOS hospital [days]** | 1819 | 0.1 | (0.1 - 0.3) | 0.1 | (0.1 - 0.2) | 0.1 | (0.1 - 0.3) | 0.773 | 1.016 | (0.979;1.054) | | 0.410 | |
| **In-hospital death** | 1819 | 4 | (0.2) | 2 | (0.1) | 2 | (1.0) | 0.010 | 8.503 | (1.191;60.707) | | **0.033** | |
| **Total cost ED [Swissfrancs]** | 1819 | 714 | (447 - 1222) | 715 | (447 - 1229) | 695 | (424 - 1176) | 0.562 | 1.000 | (1.000;1.000) | | **0.035** | |
| **Total cost hospital [Swissfrancs]** | 1819 | 770 | (476 - 1484) | 766 | (474 - 1481) | 792 | (530 - 1511) | 0.426 | 1.000 | (1.000;1.000) | | **0.012** | |

Categorical variables are shown with number (%) in each category, p-values obtained by Chi-squared test.

Continous variables are described with median (IQR), p-values obtained by Wilcoxon rank sum test.

* bold = p-value beneath statistical significance level (< 0.05). italic = p-value at least weak association (< 0.2)

**Supplement table 2 :** Comparison to comparison group (young adults 19-25 years old) in the validation set using logistic regression analysis.
