# Supplementary material for: Emergency department utilisation and treatment for trauma-related presentations of adolescents aged 16–18: a retrospective cross-sectional study
Source: BMC Emerg Med. 2024 Feb 27;24:33. doi: 10.1186/s12873-024-00945-8 (PMC10900568; doi:10.1186/s12873-024-00945-8)
Supplement: Supplementary file 3 — Supplement Table 3: Multivariable analysis for the comparison group [file 12873_2024_945_MOESM3_ESM.docx]

|  | **Odds Ratio** |  |  |
| --- | --- | --- | --- |
| **Treatment operation** | **Adjusted Odds ratio** | **(95% CI)** | **p-value** |
| **Sex** |  |  |  |
| Female | 1.00 |  |  |
| Male | 3.23 | (1.22; 8.53) | 0.018 |
| **Night Shift (10pm to 6 am)** | 0.30 | (0.09; 0.96) | 0.042 |
| **Documented high urgency** | 2.16 | (0.83; 5.65) | 0.115 |
| **Documented walk-in** | 0.21 | (0.09; 0.49) | <0.001 |
| **Upper extremity** | 2.81 | (1.21; 6.52) | 0.016 |
| **Violence** | 2.98 | (0.87; 10.24) | 0.083 |
| A total of 193 observations were included in the final model. | |  |  |
| AUROC = 0.803. |  |  |  |

**Supplement table 3:** Multivariable analysis for the comparison group.
